# User Archetypes of a Well-Being–Promoting Mobile App Among Adults: Cross-Sectional Study and Cluster Analysis of Usage Patterns

**DOI:** 10.2196/68982

**Published:** 2025-08-18

**Authors:** Hanna Rekola, Tommi Tolmunen, Elina Mattila, Juho Strömmer, Timo A Lakka, Helena Länsimies, Tomi Mäki-Opas

**Affiliations:** 1 Department of Social Sciences, Faculty of Social Sciences and Business Studies, University of Eastern Finland, PO Box 1627, Kuopio, 70211, Finland, 358 469214359; 2 Social, Wellbeing, and Rescue Research Centre, Wellbeing Services County of North Savo, Kuopio, Finland; 3 Institute of Clinical Medicine, School of Medicine, Faculty of Health Sciences, University of Eastern Finland, Kuopio, Finland; 4 Department of Adolescent Psychiatry, Kuopio University Hospital, Kuopio, Finland; 5 VTT Technical Research Centre of Finland Ltd, Espoo, Finland; 6 Wellpro Impact Solutions Oy, Jyväskylä, Finland; 7 Institute of Biomedicine, School of Medicine, Faculty of Health Sciences, University of Eastern Finland, Kuopio, Finland; 8 Department of Clinical Physiology and Nuclear Medicine, Kuopio University Hospital, Kuopio, Finland; 9 Foundation for Research in Health Exercise and Nutrition, Kuopio Research Institute of Excercise Medicine, Kuopio, Finland; 10 City of Kuopio, Kuopio, Finland

**Keywords:** user archetypes, adherence, user characteristics, digital well-being promotion, mobile app, lifestyle, behavior modification, behavioral change

## Abstract

**Background:**

A healthy lifestyle is associated with mental well-being, and digital lifestyle interventions can be effective in promoting a healthy lifestyle. However, they do not appear to work for all, and we have limited knowledge of how users’ background characteristics affect their tendency to adopt well-being–promoting digital apps and actively use them.

**Objective:**

This study aimed to explore the association of the study participants’ characteristics and current well-being with their likelihood of using a well-being–promoting mobile app.

**Methods:**

The BitHabit web app (Wellpro Impact Solutions Ltd) was available for a 2-month trial in spring 2023 after completing a short cross-sectional digital questionnaire with questions about well-being, life satisfaction, and lifestyle. Individuals aged 15 years or younger were excluded from the analysis. We used logistic regression to assess how individual characteristics were associated with the initiation of BitHabit app use. To assess user archetypes among those who initiated app use, and k-means clustering analysis and multinomial logistic regression to assess user archetypes among those who initiated app use.

**Results:**

A total of 1646 eligible individuals responded to the questionnaire, and 863 initiated app use. Lower odds of initiating app use were detected among males (odds ratio [OR] 0.66, 95% CI 0.51‐0.85; *P*<.001), the unemployed (OR 0.68, 95% CI 0.48‐0.97; *P*=.03), those with higher general life satisfaction (OR 0.94, 95% CI 0.89‐1.00; *P*=.04), and those reporting fewer life challenges (OR 1.13, 95% CI 1.02‐1.24; *P=*.02). We identified (1) thriving non-active users, (2) struggling non-active users, and (3) active users as archetypes based on app use activity, life satisfaction, and reported life challenges. Older participants had lower odds of being thriving nonactive (OR 0.96, 95% CI 0.94‐0.99; *P*=.01) or struggling nonactive users (OR 0.93, 95% CI 0.90‐0.96; *P*<.001) than active users. Retired participants had higher odds of being struggling nonactive than active users (OR 4.06, 95% CI 1.44‐11.42; *P*=.01) and unemployed lower odds of being thriving nonactive than active users (OR 0.2, 95% CI 0.08‐0.51; *P*<.001). Those who were physically more active had higher odds of being thriving nonactive than active users (OR 2.71, 95% CI 1.00‐7.32; *P*=.05). Participants with higher alcohol consumption had higher odds of being struggling nonactive users than active users (OR 3.22, 95% CI 1.16‐8.99; *P*=.03).

**Conclusions:**

While lower general life satisfaction and less favorable health behavior appeared to increase the likelihood of trying the app, those who eventually actively used the app were more satisfied with their lives at baseline. In addition, among nonactive users, there were recognizable user profiles of thriving and struggling nonactive users, which were associated with various individual characteristics. Further research is needed to develop digital apps to attract more potential users and meet the needs of those with an unhealthy lifestyle and poor mental health.

## Introduction

Mental health challenges pose a burden on well-being and public health, partly due to digitalization and changes in lifestyle and working life [1]. On the other hand, in social and health services, new digital apps and innovations are becoming increasingly common, and the promotion of health and well-being is becoming more important than ever. Digital interventions (ie, eHealth and mobile health [mHealth] tools) might have the potential to tackle these challenges.

Based on extensive evidence, a healthy lifestyle is associated with mental health and well-being [1]. Digital lifestyle interventions are effective in promoting not only a healthy lifestyle but also mental health [2-4]. More specifically, empirical evidence supports the effectiveness of lifestyle modification based on self-determination theory [5] and a self-regulation approach [6]. However, little research has been conducted on digital well-being–promoting interventions in healthy populations [4]. Furthermore, these interventions do not appear to produce the same outcomes for everyone, and there is still limited evidence on how user characteristics affect the tendency to adopt digital apps to promote well-being and how people use these apps [7].

**Figure 1.**
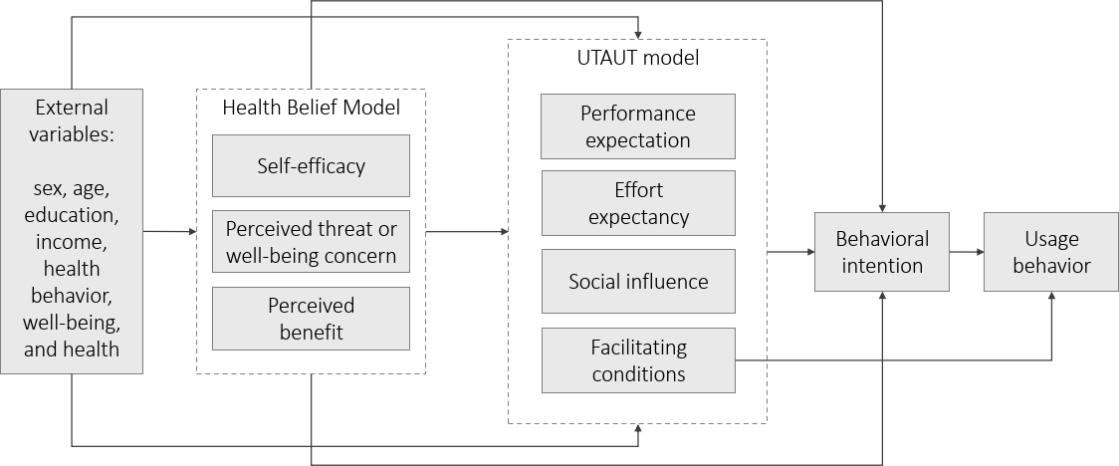
The combined theoretical framework of the Health Belief Model and the Unified Theory of Acceptance and Use of Technology. UTAUT: Unified Theory of Acceptance and Use of Technology.

We use a combination of health behavior (the Health Belief Model [HBM]) [8] and technology acceptance perspectives (the Unified Theory of Acceptance and Use of Technology [UTAUT]) [9] to understand the mechanisms underlying the selection and adoption of well-being–promoting apps (Figure 1). The HBM suggests that perceived susceptibility to health issues and perceived benefits of action influence individuals’ motivation to adopt preventive strategies, such as well-being apps. In addition, users with high self-efficacy, or confidence in their ability to manage their well-being, may also be more likely to use the app. Based on the UTAUT model, app engagement is more likely when users expect it to improve their well-being, find it easy to use, perceive social endorsement, and have adequate access to technology. In this context, demographic and psychosocial factors may shape how likely individuals are to engage with well-being–promoting apps.

According to existing evidence, women are more likely to seek health information on the internet compared with men [10], and women also appear to be more willing to adopt digital tools to promote their health and well-being [11,12], but contradictory results have been obtained in some studies [13]. In addition, among working-age adults, older individuals have been found to use digital well-being–promoting apps more frequently than younger people [11-15]. Social determinants, such as employment, education, or income, have been associated in some studies with the use of digital health promotion tools, but the results have also been contradictory [11-13,16]. While in some studies, the unemployed have been more likely to use digital health interventions as recommended [11], in others, they have been less likely to initiate use of a digital lifestyle intervention [13].

It has been suggested that digital well-being–promoting interventions are particularly attractive to people with a greater need for health information. Some evidence suggests that people with lower life satisfaction, an unhealthy lifestyle, health issues, and challenges are more likely to initiate the use of digital health–promoting apps and use them more frequently compared with the general population [7,13,17]. One reason behind this attraction could be the perceived anonymity behind digital interventions, which might make them more appealing, for example, to people with a lower level of physical activity, poorer health, and mental health problems [18,19]. Conversely, some researchers have proposed that behavioral intervention programs may better reach those who already have healthier lifestyles, higher life satisfaction, and better well-being at baseline [11,14,15,20], and who thus need these interventions the least.

This study was a part of the Feeling Good North Savo program [21]. The aim of the study was to explore the associations between the characteristics of participants and their likelihood of using a mobile app promoting well-being. Our assumption, based on the limited existing evidence, was that women, older people, and employed people—indicating a higher socioeconomic status—would be more interested in trying and using the app. In addition, we expected that people with healthier lifestyles, fewer reported life challenges, and better life satisfaction would be more likely to use the app.

Our specific research questions were: (1) Are individual characteristics, behavioral factors, and the well-being of the participants associated with the probability of initiating the use of a well-being–promoting app? and (2) How do user archetypes, identified based on activity in using the app, reported life challenges, and life satisfaction, differ in terms of individual characteristics, behavioral factors, and well-being among the participants who initiated app use?

## Methods

### Study Design and Recruitment

This observational cross-sectional study is reported following the STROBE (Strengthening the Reporting of Observational Studies in Epidemiology) [22] (the STROBE checklist is provided in Checklist 1) and mERA (mHealth Evidence Reporting and Assessment) [23] guidelines (the mERA checklist is provided in Checklist 2).

The web-based questionnaire and intervention were available to the public from late February until early May 2023. The study was promoted in the North Savo region of Eastern Finland through local newspapers, municipalities’ communication channels, and the Feeling Good North Savo program’s social media and website. Everyone who came across the invitation could participate in the study. We had no definitive inclusion or exclusion criteria, as the study was designed to act as a feasibility study for a further definitive trial. However, due to ethical reasons, one individual under the age of 15 years was excluded from the analysis. In addition, as the study invitation, digital questionnaire, and intervention were conducted in Finnish, proficiency in the Finnish language was an implicit inclusion criterion. Completing the MySeula digital questionnaire took approximately 5 minutes. Afterward, participants had the option to register for the BitHabit app and use it for 1-2 months, depending on when they joined the study.

### BitHabit Web App

Our intervention model used the MySeula digital questionnaire and the BitHabit web application provided by Wellpro Impact Solutions [24]. The MySeula digital survey was used to collect the study data, including background characteristics as well as well-being and lifestyle information. Each time participants completed the survey, they received an automatic report on their well-being status. In addition to collecting the study data, the digital survey was intended to steer the use of the BitHabit web app and raise awareness of well-being promotion among the participants.

The BitHabit web app was originally developed in the Finnish Stop Diabetes study to motivate and facilitate adults to improve their health behavior by providing valuable information and recommending small habits to be integrated into daily routines [2,25]. The BitHabit web app combined with group meetings was found effective in improving the diet quality of the study participants [2]. In addition, a web app without the group meetings was associated with increased physical activity and decreased sedentary time, but only among participants with good adherence to the intervention.

The principal concept of the app is based on self-determination behavior change theory, which supports autonomous motivation and competence, emphasizing freedom of choice and drawing on users’ existing knowledge, skills, and habits [5]. Furthermore, the app relies on the habit formation theory, which promotes behavior change through repetition of simple, contextualized, and frequent behaviors to achieve habit automaticity [26-28]. In addition, app development is based on behavior change techniques from self-regulation theory, such as self-monitoring, goal setting, and action planning [6,29].

BitHabit is a web application accessible from any modern browser on smartphones and computers. Infrastructure to support technology operations in North Savo is well developed, with widespread internet access and networks. The app user has access to a comprehensive library of well-being–promoting habits, as described in detail earlier [25]. The application is based on the idea that users engage with it on their own initiative: the user explores the library of habits, plans how to integrate them into their daily life based on their personal needs and the results of the MySeula survey, and logs completed habits in the application. Users can monitor their progress by keeping track of the habits they have completed. The app sends prompts to the user via text message. These prompts are generic reminders to use the app, particularly when it has not been used for a while. The application does not directly suggest specific small actions to the user.

In this study, the app was iteratively tested by trained experts to better fit the target group’s needs. Across 3 rounds with 8 users, technical and content adjustments were made, including the addition of several mental health–related daily habits. These included relaxation and breathing exercises, mindfulness, fostering positive self-talk, and writing down personal thoughts, among others.

### Measures

#### Behavioral Factors

Physical activity of the study participants was measured using questions adapted from the Stop Diabetes study [2], the Finnish National FINRISK Study [30], and the International Physical Activity Questionnaire [31]. We inquired with structural questions about how many hours per week the participants carried out conditioning physical activity. We also inquired, with a structural question, how many hours the respondents engaged in light physical activity or spent sitting or lying during a day (excluding sleeping). The satisfaction of participants with their current physical condition was also assessed with a self-reported measure on a scale from 1 to 10.

Alcohol consumption was assessed with a self-reported measure of how often the participants had consumed 6 or more drinks on one occasion during the past year. The item is included in the Alcohol Use Disorders Identification Test-Concise (AUDIT-C), developed by the World Health Organization (WHO) [32]. For our short questionnaire, we exclusively used the item assessing the frequency of habitual intoxication-seeking behavior.

#### Well-Being

Life satisfaction was assessed using a self-reported measure of general satisfaction with life at the moment on a scale from 1 to 10, based on the Organisation for Economic Co-operation and Development (OECD) Better Life Index. Life satisfaction subjectively measures how people evaluate their life as a whole rather than their current feelings [33]. We also assessed, with a similar scale, how satisfied the participants were with their physical condition and social relationships.

Life challenges were assessed using a structured multiple-choice question. Possible response alternatives were physical impairment, psychological impairment, cognitive impairment, difficulty in social interaction, financial challenges, or none of these. All of these were used in the analysis as binary variables. A summary variable indicating the number of life challenges reported by the participant was also computed.

#### Participant Characteristics

We included questions about age, gender, and employment status as individual characteristics. These variables were used to describe the study sample and explore subgroup differences.

#### BitHabit App Use

BitHabit app use was assessed with comprehensive user log data. All events in the log data included timestamps. For this study, we analyzed the initiation of app use, frequency of sessions, and the selection and performance of habits. “Initiation of app use” refers to the participant creating an account in the app. A “session” refers to the user launching the mobile app on their device. We calculated an “active days ratio,” which is the ratio of days with sessions to all days during the time of study participation. Performing a habit refers to the user marking a specific habit as selected or completed in the app. We also calculated the “habit ratio,” referring to the average number of habits selected or completed per day during the user’s participation in the study.

### Statistical Analysis

The data were processed and analyzed using RStudio software (R Core Team, Posit Team) [34,35]. To assess the association between the initiation of app use and user characteristics, simple unweighted 2-way crosstab tables were generated with the chi-square test of association as well as correlation matrices. For continuous variables, we tested mean differences using the Mann-Whitney *U* test. We also tested logistic regression models that included the background characteristics at the same time, accounting for dependencies among the variables to better understand their influence on the initiation of app use. We included in the logistic regression models all confounders analyzed in the univariate assessments, with two exceptions: only the physical activity indicator with the highest statistical significance was retained, and only general life satisfaction was included due to its high correlation with satisfaction in social relationships and physical condition. Missing information is presented in the crosstabulation tables and excluded from the logistic regression models. A cutoff of *P*<.05 was used to determine statistical significance in all analyses.

Different user archetypes among participants who initiated app use were recognized using k-means clustering analysis. The methodology was similar to that of Aziz et al [36]. The k-means clustering method defines clusters so that the total within-cluster variation is minimized. The “kmeans” function from the R package “stats” [35] was used with the default clustering algorithm by Hartigan and Wong [37]. In the Hartigan-Wong algorithm, each participant is assigned to a cluster so that the sum of squared Euclidean distances between observations and the cluster centroid, that is, the total within-cluster variation, is minimized. For the visualizations, we used the R packages “factoextra” [38] and “ggplot2” [39].

The clustering analysis included 4 variables: general life satisfaction, number of reported life challenges or impairments, ratio of active app usage days to no usage days, and average number of reported habits per day. The clustering variables were chosen based on the findings from previous studies indicating that the number of health issues and life satisfaction would be associated with app usage patterns, but with conflicting results [7,11,13-15,17-20].

**Figure 2.**
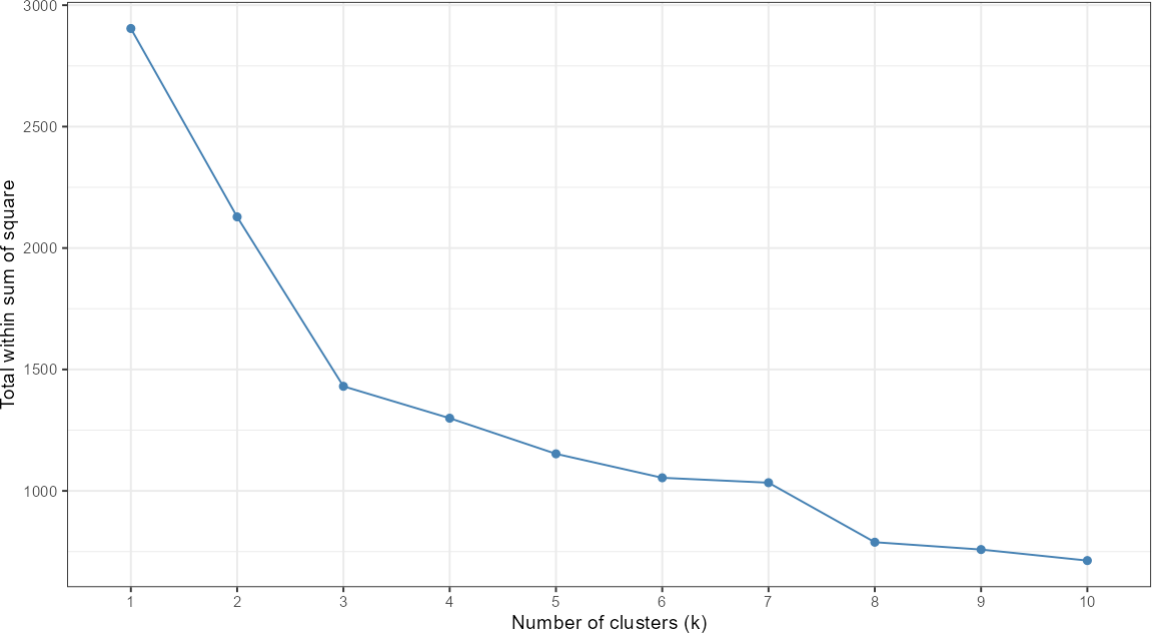
Elbow plot of the optimal number of clusters among participants who initiated BitHabit app use.

The optimal number of clusters was determined using the elbow method, which assessed the total within-cluster sum of squares across solutions with 2 to 10 clusters (Figure 2). The plot showed a clear inflection point at k=3, beyond which additional clusters yielded only marginal improvements in model fit. This was supplemented by manual inspection of cluster interpretability with models based on 2-5 clusters, leading to the selection of 3 clusters. One of the clusters accounted for 65% (474/727) of users, and the smallest 8% (55/727). The unequal cluster size distribution was anticipated based on our hypotheses and characteristics of the sample. It was expected that a substantial proportion of participants would demonstrate low engagement with the app, reflecting typical patterns in previous studies [2].

**Figure 3.**
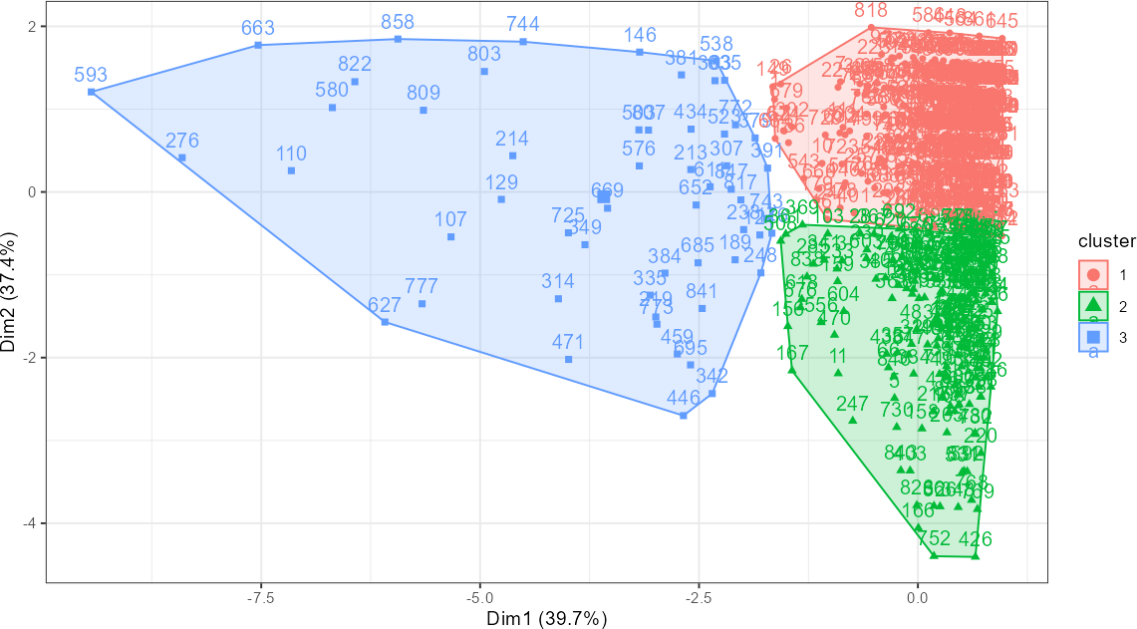
Principal component analysis plot colored by cluster assignment.

To further assess the classification bias, we examined the principal component analysis plots by cluster color to assess separation and dominance patterns and detected no overlapping (Figure 3). We also assessed the stability of the clustering by executing the algorithm several times with different random initializations, and the results remained stable across runs. Finally, we used univariate assessments and multinomial logistic regression to assess how different user characteristics were represented among the clusters. The confounders included in the multinomial regression model were the same as those used in the logistic regression model for app use initiation, excluding the variables involved in the clustering analysis.

### Ethical Considerations

The University of Eastern Finland is committed to adhering to the ethical guidelines set forth by the Finnish National Board on Research Integrity (TENK) [40]. These guidelines apply to research involving human participants, except for medical research as defined by the Finnish Medical Research Act (488/1999) [41] and other study types for which ethical review is mandated separately by law. Following TENK’s principles, research must be conducted in a manner that upholds the dignity and autonomy of participants and avoids causing significant risk, harm, or damage to individuals, communities, or other participants of research.

An ethical review must be sought before data collection if the study includes any of the following conditions: (1) participation in the study deviates from the principle of informed consent, such as when participation is not voluntary or when participants are not adequately informed about the research; (2) the research involves interventions that affect the physical integrity of participants; (3) the study focuses on children aged 15 years or younger without obtaining separate consent from a parent or guardian, or without providing them with sufficient information to allow them to prevent the child’s participation; (4) participants are exposed to exceptionally strong stimuli; (5) the research carries a risk of causing mental harm beyond the typical stresses of daily life to participants, their family members, or others close to them; and (6) the research may pose a threat to the safety of participants, researchers, or their close contacts. None of these conditions were met in this study; hence, an ethical review was not required.

Informed consent was considered to have been given when participants, after reading the study invitation, chose to respond to the questionnaire. The invitation stated how the collected data would be used in the study conducted by the University of Eastern Finland. It was also stated that no individual user could be identified or traced based on the data collected.

Personal data, such as email addresses, was collected by the service provider during app registration for customer management purposes. No national identification numbers or other sensitive personal data were collected. All data transfers outside the European Union and the European Economic Area, if any, were conducted following Article 46 of the General Data Protection Regulation [42], using appropriate safeguards such as the European Commission’s adequacy decisions or standard contractual clauses. The data used by the research team were deidentified, and they only had access to pseudonymized datasets that did not include any directly identifiable information. Participants received no compensation for their participation in the study.

## Results

### Descriptive Statistics

**Figure 4.**
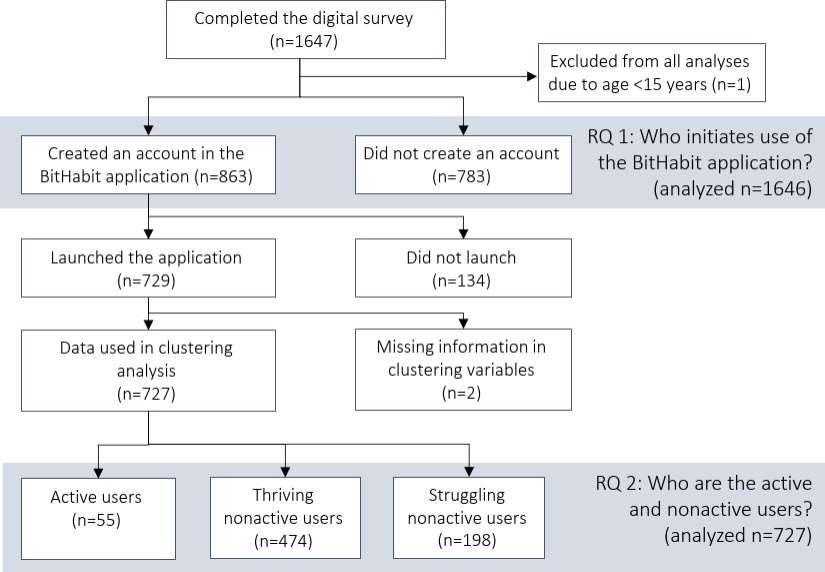
Flowchart of participant selection for the analyses. RQ: research question.

Figure 4 shows the participant flow of the study. The total number of participants was 1646 (Figure 4), and 77% (1263/1642) of those with nonmissing gender information were women. The mean age among participants was 46.7 (SD 14.2) years. The majority of the participants were used (57%, 937/1646) or retired (18%. 289/1646; Table 1). Most participants (69%, 1135/1642) reported engaging in conditioning exercise for more than 1 hour per week. Approximately 10% (182/1641) of participants consumed 6 or more doses of alcohol every week, and 23% (370/1641) monthly or more often. Almost 80% (1271/1641) reported consuming 6 or more doses less than once a month. Men reported consuming alcohol more often than women.

Participants reported having, on average, at least 1 life challenge; 34% (553/1646) of the participants did not report any. The most commonly reported life challenges were psychological impairment (31%, 516/1646) and physical impairment (29%, 477/1646). Most rarely, participants reported having cognitive impairment (17%, 273/1646). General life satisfaction among the participants was 6.6 on average.

**Table 1.** Individual characteristics of study participants (N=1646), stratified by initiation of BitHabit app use.^,^

Characteristic	All participants (N=1646)	Initiated app use (n=863)	Did not initiate app use (n=783)	*P *value^a^
Age (years), mean (SD)	46.7 (14.2)	46.5 (13.8)	46.9 (14.5)	.51
Missing, n^b^	0	0	0	
Gender, n (%)				<.001
Male	363 (22.1)	159 (18.4)	204 (26.2)	
Female	1263 (76.9)	694 (80.4)	569 (73.0)	
Other	16 (1.0)	10 (1.2)	6 (0.8)	
Missing, n	4	0	4	
Employment status, n (%)				.47
Employed	937 (56.9)	507 (58.7)	430 (54.9)	
Retired	289 (17.6)	146 (16.9)	143 (18.3)	
Student	148 (9.0)	75 (8.7)	73 (9.3)	
Entrepreneur	108 (6.6)	53 (6.1)	55 (7.0)	
Unemployed	91 (5.5)	41 (4.8)	50 (6.4)	
Other	73 (4.4)	41 (4.8)	32 (4.1)	
Missing, n	0	0	0	
Six or more doses of alcohol on one occasion, n (%)				.15
Never	526 (32.0)	267 (30.9)	259 (33.1)	
Less than once a month	745 (45.3)	412 (47.7)	333 (42.5)	
Once a month	188 (11.4)	96 (11.1)	92 (11.7)	
Once a week	158 (9.6)	71 (8.2)	87 (11.1)	
Daily or almost daily	24 (1.5)	13 (1.5)	11 (1.4)	
Missing, n	5	4	1	
Sedentary per day, n (%)				.36
5≥ hours	517 (31.4)	263 (30.5)	254 (32.4)	
6-8 hours	747 (45.4)	389 (45.1)	358 (45.7)	
>8 hours	378 (23.0)	210 (24.3)	168 (21.5)	
Missing, n	4	1	3	
Conditioning exercise per week, n (%)				.02
<1 hour	507 (30.8)	276 (32.0)	231 (29.5)	
1-3 hours	757 (46.0)	409 (47.4)	348 (44.4)	
4≤ hours	378 (23.0)	174 (20.2)	204 (26.1)	
Missing, n	4	4	0	
Light physical activity per day, n (%)				.006
<2 hours	460 (27.9)	245 (28.4)	215 (27.5)	
2-3 hours	739 (44.9)	411 (47.6)	328 (41.9)	
>3 hours	445 (27.0)	205 (23.8)	240 (30.7)	
Missing, n	2	2	0	
Life challenges or impairments, n (%)^c^				
Physical impairment	477 (29.0)	265 (30.7)	212 (27.1)	.10
Psychological impairment	516 (31.3)	296 (34.3)	220 (28.1)	.007
Cognitive impairment	273 (16.6)	159 (18.4)	114 (14.6)	.04
Difficulty in social interaction	332 (20.2)	193 (22.4)	139 (17.8)	.02
Financial challenges	394 (23.9)	218 (25.3)	176 (22.5)	.19
None of these	553 (33.6)	265 (30.7)	288 (36.8)	.009
Number of reported life challenges or impairments, mean (SD)	1.21 (1.2)	1.31 (1.2)	1.10 (1.2)	<.001
Missing, n	0	0	0	
Satisfaction with physical condition, mean (SD)	5.18 (2.6)	4.99 (2.6)	5.38 (2.5)	.002
Missing, n	3	1	2	
Satisfaction with social relationships, mean (SD)	6.70 (2.3)	6.57 (2.3)	6.84 (2.4)	.004
Missing, n	4	3	1	
General life satisfaction, mean (SD)	6.64 (2.1)	6.50 (2.1)	6.80 (2.2)	<.001
Missing, n	3	2	1	

a *P* values were calculated using the Mann-Whitney *U* test for continuous variables and the chi-square test for categorical variables.

b The “Missing” row for each variable refers to the number of participants with incomplete data for that variable. These cases were excluded from the corresponding statistical tests.

c For life challenges or impairments, each item was analyzed as a binary variable. Percentages for their frequency refer to the prevalence among participants. The chi-square test has been separately calculated for each binary variable.

### Initiation of BitHabit App Use

In total, 863 of 1646 (52%) participants initiated BitHabit app use. Based on univariate assessments (Table 1), the initiation of BitHabit app use was positively associated with female gender, less conditioning exercise and light physical activity per week, psychological impairment, cognitive impairment, difficulty in social interaction, a higher number of reported life challenges or impairments, lower satisfaction with physical condition and social relationships, as well as lower general life satisfaction. Furthermore, initiation of BitHabit app use was negatively associated with not reporting any of the listed life challenges or impairments.

Men were also less likely than women to start using the BitHabit app based on the logistic regression model (Table 2; odds ratio [OR] 0.66, 95% CI 0.51‐0.85). This association was persistent in all the models generated, indicating that gender has a strong independent effect on the initiation of BitHabit app use, even after accounting for other variables in the data.

**Table 2.** Logistic regression of determinants for initiation of app use compared with not initiating (N=1614 due to missingness in independent variables).

Covariate	OR^a^ (95% CI)	*P *value
Intercept	1.64 (0.89-3.02)	.11
Male (ref: female)	0.66 (0.51-0.85)	<.001
Age (years)	1.00 (0.99-1.01)	.76
Retired (ref: employed or entrepreneur)	0.85 (0.61-1.17)	.32
Student (ref: employed or entrepreneur)	0.81 (0.55-1.19)	.28
Unemployed, full or part-time disability pension, or other situation (ref: employed or entrepreneur)	0.68 (0.48-0.97)	.03
Conditioning exercise 1‐3 hrs/wk (ref: <1)	1.03 (0.82-1.31)	.79
Conditioning exercise ≥4 hrs/wk (ref: <1)	0.86 (0.64-1.15)	.30
Consumes ≥6 doses of alcohol on one occasion monthly or more frequently (ref: no)	0.91 (0.71-1.16)	.44
General life satisfaction	0.94 (0.89-1.00)	.04
Number of reported life challenges or impairments	1.13 (1.02-1.24)	.02

a OR: odds ratio comparing the likelihood of initiating app use relative to not initiating.

When age was assessed as a continuous variable in the logistic regression model, no statistically significant difference was detected (Table 2). When differences were assessed separately according to age groups, we found in univariate assessments that participants aged 30-50 years were somewhat more likely to start using BitHabit, but these differences were also statistically nonsignificant.

Employment status was not associated with the initiation of BitHabit app use in the univariate assessments, but in the logistic regression model, unemployed participants had lower odds (Table 2; OR 0.68, 95% CI 0.48‐0.97) of initiating app use than employed participants or entrepreneurs. A student status or retirement did not predict the initiation of app use.

Even though physical activity was negatively associated with the initiation of BitHabit app use in the univariate assessments, no such associations were found in the logistic regression model. Furthermore, alcohol use was not associated with the initiation of app use in either univariate assessments or the logistic regression model.

Participants who were less satisfied with some aspect of their life appeared to be more likely to start using the BitHabit app (Table 1 and Table 2). Those with higher general life satisfaction had lower odds of initiating app use (Table 2; OR 0.94, 95% CI 0.89‐1.00). In addition, a higher number of reported life challenges or impairments was associated with increased odds of initiating app use (Table 2; OR 1.13, 95% CI 1.02‐1.24).

### User Archetypes of the BitHabit App

**Figure 5.**
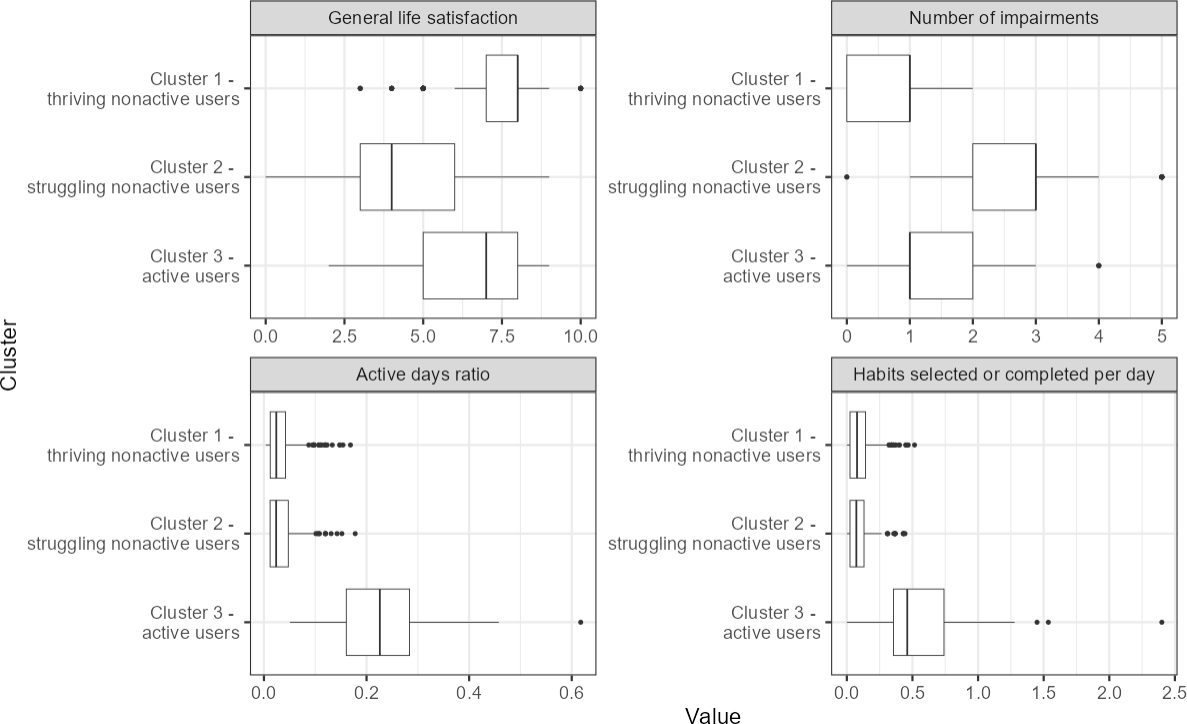
Boxplot of clustering variables for the different user archetype clusters among participants. The horizontal line represents the median, the box hinges represent the first and third quartiles, and whiskers extend from the hinge to the largest and smallest values, but not further than 1.5 times the IQR from the hinge.

In the clustering analysis for participants who launched the BitHabit app, we recognized 3 different user archetypes based on general life satisfaction, the number of reported impairments or life challenges, the active days ratio (proportion of days with sessions in the app), and the habits ratio (number of habits selected or completed per day). We labeled these archetypes as “thriving nonactive users” (n=474), “struggling nonactive users” (n=198), and “active users” (n=55; Figure 5). In the first cluster, participants had high life satisfaction and a small average number of impairments or life challenges, and they used the app infrequently. In the second cluster, on the other hand, participants had significantly lower life satisfaction and multiple reported impairments on average, but they still used the app infrequently. The struggling nonactive users especially reported financial difficulties (57%, 113/198) and psychological impairment (77%, 153/198; Table 3). In the third cluster, the participants’ life satisfaction and reported number of impairments were at similar levels as in cluster 1, but they used the BitHabit app more frequently. In other words, the participants who actively used the app were thriving at baseline, but among nonactive users, we could recognize participants who were thriving and those who were struggling.

Based on univariate descriptive statistics (Table 3) and the multinomial regression model (Table 4), individual characteristics and behavioral factors were associated with the participants’ user archetype. Compared with active users, thriving nonactive users were more often younger participants, employed or entrepreneurs, engaged in more physical activity, and they reported fewer life challenges. Struggling nonactive users were also more often younger participants, retired, engaged in less physical activity, and consumed alcohol more often. The struggling nonactive users, in particular, reported cognitive impairment, and very few of them reported physical impairment (Table 3).

**Table 3.** Individual characteristics of participants who initiated use of the BitHabit app (N=727), stratified by user archetype.^,^

Characteristic	All participants who initiated app use (N=727)	Thriving nonactive users (n=474)	Struggling nonactive users (n=198)	Active users (n=55)	*P* value^a^
Age (years), mean (SD)	46.3 (13.3)	47.2 (13.5)	42.9 (12.5)	51.0 (12.7)	<.001
Missing, n^b^	0	0	0	0	
Gender, n (%)					.11
Male	127 (17.5)	79 (16.7)	41 (20.7)	7 (12.7)	
Female	591 (81.3)	392 (82.7)	153 (77.3)	46 (83.6)	
Other	9 (1.2)	3 (0.6)	4 (2.0)	2 (3.6)	
Missing, n	0	0	0	0	
Employment status, n (%)					<.001
Employed or entrepreneur	479 (65.9)	349 (73.6)	98 (49.5)	32 (58.2)	
Retired	116 (16.0)	74 (15.6)	32 (16.2)	10 (18.2)	
Student	62 (8.5)	34 (7.2)	24 (12.1)	4 (7.3)	
Unemployed, full or part-time disability pension, or other situation	70 (9.6)	17 (3.6)	44 (22.2)	9 (16.4)	
Missing, n	0	0	0	0	
Six or more doses of alcohol on one occasion, n (%)					.007
Less than once a month or never	585 (80.7)	388 (82.2)	147 (74.2)	50 (90.9)	
At least once a month	140 (19.3)	84 (17.8)	51 (25.8)	5 (9.1)	
Missing, n	2	2	0	0	
Sedentary per day, n (%)					.006
5 hours or less	216 (29.8)	143 (30.2)	50 (25.3)	23 (41.8)	
6 to 8 hours	331 (45.6)	228 (48.2)	82 (41.4)	21 (38.2)	
More than 8 hours	179 (24.7)	102 (21.6)	66 (33.3)	11 (20.0)	
Missing, n	1	1	0	0	
Conditioning exercise per week, n (%)					<.001
Less than 1 hour	236 (32.6)	121 (25.7)	98 (49.5)	17 (30.9)	
1 to 3 hours	342 (47.2)	231 (49.0)	79 (39.9)	32 (58.2)	
4 or more hours	146 (20.2)	119 (25.3)	21 (10.6)	6 (10.9)	
Missing, n	3	3	0	0	
Light physical activity per day, n (%)					.06
Less than 2 hours	205 (28.2)	120 (25.4)	70 (35.4)	15 (27.3)	
2 to 3 hours	352 (48.5)	241 (51.0)	88 (44.4)	23 (41.8)	
More than 3 hours	169 (23.3)	112 (23.7)	40 (20.2)	17 (30.9)	
Missing, n	1	1	0	0	
Life challenges or impairments, n (%)^**c**^					
Physical impairment	227 (31.2)	107 (22.6)	95 (48.0)	25 (45.5)	<.001
Psychological impairment	250 (34.4)	77 (16.2)	153 (77.3)	20 (36.4)	<.001
Cognitive impairment	129 (17.7)	42 (8.9)	73 (36.9)	14 (25.5)	<.001
Difficulty in social interaction	156 (21.5)	43 (9.1)	97 (49.0)	16 (29.1)	<.001
Financial challenges	171 (23.5)	45 (9.5)	113 (57.1)	13 (23.6)	<.001
None of these	229 (31.5)	213 (44.9)	3 (1.5)	13 (23.6)	<.001
Missing, n	0	0	0	0	
Number of reported life challenges or impairments, mean (SD)	1.28 (1.2)	0.66 (0.7)	2.68 (1.1)	1.60 (1.2)	<.001
Missing, n	0	0	0	0	
Satisfaction with physical condition, mean (SD)	5.01 (2.6)	5.66 (2.3)	3.44 (2.6)	5.13 (2.4)	<.001
Missing, n	1	1	0	0	
Satisfaction with social relationships, mean (SD)	6.11 (2.4)	6.53 (2.1)	5.15 (2.8)	6.00 (2.2)	<.001
Missing, n	1	1	0	0	
General life satisfaction, mean (SD)	6.55 (2.1)	7.49 (1.2)	4.34 (2.0)	6.42 (1.9)	<.001
Missing, n	0	0	0	0	

a *P* values were calculated with the Mann-Whitney *U* test for continuous variables and the chi-square test for categorical variables. The Fisher exact test was used when the assumptions of the chi-square test were not met.

b The “Missing” row for each variable refers to the number of cases with incomplete data for that variable. These cases were excluded from statistical tests.

c For the life challenges or impairments, each item was analyzed as a binary variable, and the percentages for their frequency refer to the prevalence among participants. The chi-square test was calculated separately for each binary variable.

**Table 4.** Multinomial logistic regression of predictors for user archetype clusters (N=713 due to missingness in independent variables).

Covariate	Thriving nonactive users vs active users, OR^a^ (95% CI)	*P* value	Struggling nonactive users vs active users, OR (95% CI)	*P* value
Intercept	47.55 (10.23-220.92)	<.001	97.08 (19.28-488.87)	<.001
Male (ref: female)	0.91 (0.37-2.22)	.84	1.55 (0.6-4.01)	.36
Age (years)	0.96 (0.94-0.99)	.01	0.93 (0.9-0.96)	<.001
Retired (ref: employed or entrepreneur)	1.38 (0.53-3.56)	.51	4.06 (1.44-11.42)	.008
Student (ref: employed or entrepreneur)	0.71 (0.2-2.57)	.60	0.93 (0.24-3.59)	.92
Unemployed, full or part-time disability pension, or another disability pension situation (ref: employed or entrepreneur)	0.2 (0.08-0.51)	<.001	1.82 (0.74-4.45)	.19
Conditioning exercise 1‐3 hrs/wk (ref: <1)	0.99 (0.52-1.91)	.99	0.4 (0.2-0.81)	.01
Conditioning exercise ≥4 hrs/wk (ref: <1)	2.71 (1.00-7.32)	.049	0.56 (0.19-1.67)	.30
Consumes ≥six doses of alcohol on one occasion monthly or more frequently (ref: no)	1.94 (0.72-5.21)	.19	3.22 (1.16-8.99)	.03

a OR: odds ratio comparing the likelihood of initiating app use relative to not initiating.

Although female gender was associated with a higher probability of initiating app use, gender was not associated with whether participants were thriving or struggling nonactive users compared with active users. Older participants had higher odds of being active users compared with both thriving and struggling nonactive users (Table 4).

Compared with employed participants or entrepreneurs, unemployed participants had lower odds of being thriving nonactive users compared with active users, but there was no significant association with whether participants were struggling nonactive users compared with active users. Retired people had higher odds of being struggling nonactive users compared with active users, but retirement was not associated with being a thriving nonactive user compared with an active user. Student status did not predict the user archetype for any of the clusters (Table 4).

High engagement in conditioning exercise was associated with participants being thriving nonactive users compared with active users. Participants engaging in 4 or more hours of conditioning exercise per week had the highest odds of being thriving nonactive users (Table 4; OR 2.7, 95% CI 1.00‐7.32). Similarly, participants engaging in 1‐3 hours of exercise per week compared with less than 1 hour had lower odds of being struggling nonactive users compared with active users (Table 4; OR 0.4, 95% CI 0.2‐0.81). 

Participants consuming 6 or more doses of alcohol on 1 occasion monthly or more frequently compared with those who did not have higher odds of being struggling nonactive users compared with active users. There was no association with participants being thriving nonactive users compared with active users.

## Discussion

### Principal Findings

Based on this study, individual characteristics, behavioral factors, and the well-being of the participants were associated with the probability of starting to use the well-being–promoting app BitHabit. In particular, we found that initiation of BitHabit app use was positively associated with female gender, being employed, lower general life satisfaction, and a higher number of reported life challenges.

We also identified 3 separate user archetypes based on app usage, life satisfaction, and reported life challenges. Generally, active users of the app were thriving at the baseline, but among nonactive users, we could recognize distinct thriving and struggling user archetypes. The probability of the participants belonging to the different user archetypes was associated with user characteristics. Older participants appeared to be more likely to be active users, but retired participants were more likely to be struggling nonactive users than active users. Unemployed participants were more likely to be active users than thriving nonusers. Furthermore, physically active participants were more likely to be thriving nonactive users or active users, and alcohol consumption appeared to be more common among struggling nonactive users.

### Comparison With Previous Work

Based on our results, women were more likely to initiate use of the BitHabit app compared to men. However, we detected no associations between gender and user archetypes. Previous evidence suggests that women are more likely to use digital tools to promote their health and well-being [10,11]. Our results are consistent in this regard for the initiation of app use, but not for actual use of the app. In a previous study assessing the user trajectories of the BitHabit app [14], we also detected no gender differences with regard to activity in using the app. However, the lack of statistical significance in this study could be due to the small number of both men and active users among our participants.

In our study, age was not associated with the probability of initiating use of the BitHabit app, but it was associated with the user archetype. After the initiation of app use, older participants were more likely to be active users compared to both struggling and thriving nonactive users. In other words, older participants with higher life satisfaction were more likely to be active users than to find the app unnecessary and dismiss it. This is partly consistent with existing evidence suggesting that older individuals are likely to initiate use of digital well-being–promoting apps and use them more frequently compared to younger people [11,13].

Regarding social determinants, we found that unemployed participants were less likely to initiate BitHabit app use compared with employed participants. Unemployed participants were more likely to be active users than thriving nonactive users. However, there was no association with whether unemployed participants were more likely to be struggling nonactive users than active users, indicating that it is equally likely for an unemployed participant to belong in either user archetype. Previous evidence on the participant is unclear, but our findings support the assumption that the unemployed are less likely to initiate the use of well-being–promoting digital interventions [13]. However, based on our results, after the initiation of app use, there appears to be some potential for the unemployed to continue using. Unemployed people generally appear to have lower life satisfaction and more life challenges and impairments compared with employed people [43].

Retirement was not associated with the probability of starting to use the BitHabit app, but in the user archetype analysis, retired participants were more likely to be struggling nonactive users than active users. Retired participants were more often aged 65 years or older compared with employed participants, so this finding is inconsistent with the results regarding participant age. Furthermore, no associations were found for students with regard to either the initiation of app use or the user archetype analysis.

General life satisfaction among the study participants, measured with the life satisfaction item of the OECD Better Life Index [33], was 6.6. This is lower than the average of 8.1 in Finland, which ranks highest among the OECD member countries and is much higher than the OECD average of 7.4 in 2018 [44]. Our results indicate that those who initiated app use were generally less satisfied with their life and reported more life challenges and impairments. Based on existing evidence, people with an unhealthy lifestyle and health issues would be more likely to initiate the use of digital health promotion apps [7,13,17], which is consistent with our findings.

However, in the user archetype analysis of our study, we found that the active users of the BitHabit app actually had relatively high life satisfaction compared with both nonuser groups. In other words, while life challenges and lower general life satisfaction appeared to increase the likelihood of trying out the BitHabit app, in the user archetype analysis, we noticed that those who started using the app more actively were initially more satisfied with their lives. Furthermore, among the nonactive users of the BitHabit app, we recognized distinct user archetypes for both thriving and struggling nonactive users. Our findings are to some extent aligned with studies proposing that behavioral intervention programs may primarily appeal to those who already have healthier lifestyles and thus need the interventions the least [11,20].

These findings can be interpreted through the lenses of the HBM [8] and the UTAUT [9]. Those experiencing lower life satisfaction and a higher number of life challenges may have perceived a higher susceptibility to health problems and greater urgency to act [8], which could explain their initial uptake of the app. However, while initial adoption may be driven by a sense of need, continued use requires the users to find the app beneficial and easy to use [9], as well as for them to have the necessary support and infrastructure to continue engaging with the app. It is possible that individuals with higher baseline well-being had more favorable perceptions in these areas, contributing to more consistent use. Sustained engagement may also depend on users’ self-efficacy [8], their confidence in their ability to improve their well-being and use the app effectively, which tends to be lower among individuals in more disadvantaged socioeconomic positions [45].

### Strengths and Limitations

Our results bring novel insights to the discussion of how people’s individual characteristics, behavioral factors, and well-being influence their likelihood of initiating the use of well-being–promoting digital apps and actively using them. The key strengths of our study include the relatively large baseline sample. We also encountered considerable interest in our digital well-being survey and report, as well as the BitHabit app.

However, due to resource and timing restrictions and the fact that our study was planned to act as a pilot study for a subsequent randomized controlled trial, our research design was not rigorous, and we allowed the sample to self-select without definitive inclusion criteria. We can assume that the sample is biased, and we are aware that the results of the study can at most be generalized to people who are already interested in their well-being and inclined to promote it.

We compared our sample with national statistics of the Finnish general adult population and found that older individuals, and especially men, were underrepresented in our self-selected sample. Most of the sample consisted of women, which needs to be considered when interpreting the results. Based on our previous results, men are more difficult to recruit in lifestyle intervention studies compared with women [19]. In addition, the proportion of unemployed participants aged 15-64 years in our sample was approximately 6% (91/1448), which is fairly close to the national unemployment rate among the economically active population aged 15-64 years of 7% (200,000/2,732,000), and the proportion unemployed among the total population aged 15-64 years of 6% (200,000/3,442,000) in Finland in 2023 [46]. In future studies, we could address sample selection bias through randomized or weighted sampling, as well as by marketing the study in a way that is more appealing to underrepresented population groups.

Only a small number of participants finally ended up actively using the app, which limited the sample size available for the user archetype analysis. We considered this limitation when interpreting the results of the analysis. With a larger sample size, we could have increased the number of analyzed user archetypes, and with more active users, we would have been able to conduct more detailed analysis of the profiles of both active and nonactive users in the current analysis.

Another limitation of this study is the lack of comprehensive and validated measures in the digital survey. This study was designed as a pilot study for a subsequent randomized controlled trial. To maximize the sample size in our self-selective study, we aimed to make the digital survey as short as possible and only included single-item questions. Validated multi-item questionnaires are generally preferred in most study fields, since the measurement reliability and validity of a single-item question is difficult to estimate [47]. However, the items used in this study have been widely used in previous studies or were relatively unambiguous, such as the self-reported life challenges or health behavior questions.

### Conclusions

Our results indicate that while lower general life satisfaction and less favorable health behavior appeared to increase the likelihood of trying the well-being–promoting digital app BitHabit, those who started using the app more actively were initially more satisfied with their lives. Our findings also suggest that among nonactive users, there are recognizable user profiles distinguishing thriving and struggling nonactive users. Individual characteristics such as gender, age, and employment appear to be variously associated with both initiating the well-being–promoting app and actively using the app. Our findings need to be verified in a future randomized controlled trial, preferably weighted by gender. In the future, it would also be relevant to assess the effectiveness of well-being–promoting digital apps for different user profiles and target groups.

## Supplementary material

10.2196/68982 Checklist 1 STROBE (Strengthening the Reporting of Observational Studies in Epidemiology) checklist.

10.2196/68982 Checklist 2 mERA (mHealth Evidence Reporting and Assessment) checklist.

## Abbreviations

AUDIT-C: Alcohol Use Disorders Identification Test-Concise
HBM: Health Belief Model
mERA: mHealth Evidence Reporting and Assessment
mHealth: mobile health
OECD: Organisation for Economic Co-operation and Development
OR: odds ratio
STROBE: Strengthening the Reporting of Observational Studies in Epidemiology
TENK: Finnish National Board on Research Integrity
UTAUT: Unified Theory of Acceptance and Use of Technology
WHO: World Health Organization
