# Supplementary material for: User Archetypes of a Well-Being–Promoting Mobile App Among Adults: Cross-Sectional Study and Cluster Analysis of Usage Patterns
Source: JMIR Mhealth Uhealth. 2025 Aug 18;13:e68982. doi: 10.2196/68982 (PMC12360720; doi:10.2196/68982)
Supplement: Checklist 2 [file mhealth-v13-e68982-s002.pdf]

The Adapted mERA Checklist

Proposal title	User Archetypes of a Well-being Promoting Mobile App Among the Adult Population: A Cross-sectional Selection and Cluster Analysis
Principal investigator(s)	Hanna Rekola

Adapted mERA Checklist item	Page number in research proposal where this information is reported	As necessary, justify why it is not reported
Infrastructure (population level)	5	
Technology platform	5	
Interoperability / Health information systems (HIS) context	NA	Integrating the intervention into existing HIS will be relevant after further effectiveness and cost-effectiveness evaluations.
Intervention delivery	5	
Intervention content and contextual adaptation	4-5	Currently, contextual adaptation is limited to wellbeing reports generated for users based on their individual results, aimed at guiding their use of the application. In future studies, the application will be further developed to enhance user-centeredness and allow greater customization.
Usability / content testing	5	
User feedback	5	
Access of individual participants	NA	At present, we are analyzing results from a short pilot study and facilitating factors will be assessed in further studies.
Cost assessment	NA	At present, we are analyzing results from a short pilot study and further cost-effectiveness assessment will be done in further stages of the study.
Adoption inputs / programme entry	5	At present user engagement is generic reminders to use the app and we plan to develop this in future versions of the intervention.
Compliance with national guidelines or regulatory statues and/or international best practices for security, collection, management, storage, and sharing	10	
Compliance with national public health guidelines or regulatory statutes	5	
Fidelity of the intervention	6-7	The app produces detailed user data that can be used for assessment of app usage.
Plan for sustainability and scale up	NA	Scaling up will be planned after further effectiveness and cost-effectiveness evaluations.

Adapted from: Agarwal S et al. (2016) Guidelines for reporting of health interventions using mobile phones: Mobile health (mHealth) Evidence reporting and assessment (mERA) checklist. BMJ;352:1–10. doi: https://doi.org/10.1136/bmj.i1174
